# Syrian Wives

**Published:** 1888-05

**Authors:** 


### SYRIAN WIVES.

There are grand women in Arabia; women of ability, keen in insight, and of wonderful capabilities. The duties of the wife of a Syrian to-day, are as follows:

She brings all the water for family use from a distant well. This is accomplished by filling immense jars and bringing them upon her head. She rises early and goes to the handmill of the village carrying corn, enough of which for the day's bread and she grinds by a slow, laborious process. This she carries home and cooks in an oven, which is made in the earth. It is a round hole, lined with oval and flat stones, and is heated by a fire built in it. When the bread is mixed with water and a little salt she removes the ashes and plasters pats of dough against the hot stones to cook. Could anything be more crude?

She cares for her children—usually a large family—and does all the rough work at intervals, while the husband calmly smokes his "argelie" or sits cross-legged upon his divan or house top, in converse with some equally hard-working member of Syrian society.

The houses are made of coarse stone, roughly hewn. The house-tops are of clay, covered with coarse gravel. In hot weather the sun bakes this mud-formed roof, and large cracks appear. The rain comes, and, as a natural consequence, the roof leaks. This is something of which the fastidious inhabitant of the Bible land does not approve. It does not add to his bodily comfort.

He remedies the difficulty—shall I tell you how? Not by any effort of his own; far from it; his wife comes, ascends to the house top, and in the drenching rain propels a roller of solid stone, backward and forward, much as we use a lawn-mower. This rolls the sun-dried cracks together and prevents the entrance of water.

These are only a few or the Syrian housewife's duties. Her reward is not in this world, surely. She cannot speak to her husband in public; she can receive no caress before his friends. She goes veiled and scantily clad. She has no time to make her own habiliments, for her hands must weave and spin and embroider artistically and abundantly for the husband and male children. In winter her feet are protected only by open wooden sandals, and drops of blood mark the way to the Syrian well. Of course this is among the lower and middle classes of society in Syria, but those who belong to a higher class are very few.

